# The League of Mercy and Its Future

**Published:** 1918-12-28

**Authors:** Herbert Warren

**Affiliations:** Pres. Magdalen College, Oxford


### THE LEAGUE OF MERCY AND ITS FUTURE.

### By Sir Herbert Warren, K.C.V.O., Pres. Magdalen College, Oxford.

Your Royal Highness, Mr. Chairman, ladies and gentlemen,—I highly appreciate the honour of having been asked to address you this afternoon. There are a certain number of things which, with your permission, I would like to say.

I feel it to be both a privilege and a responsibility to speak on behalf of the League of Mercy, and to its members. I felt it keenly the other day at the meeting in the Sheldonian Theatre at Oxford, to which reference has already been made. That, I believe, was a very successful, as it certainly was a very happy, gathering, and was largely rendered so by the presence of H.R.H. Princess Christian, who came down to take the place of the Princess Helena Victoria. She is an outstanding pioneer in nursing, in hospital work, and, indeed, in everything for which the League of Mercy stands, and her daughter is following in her footsteps most successfully.

The annual meeting of the League of Mercy must always be an important and special occasion, and to-day, when we have witnessed the beautiful ceremony, so graciously performed by H.R.H. Princess Mary, I think it comes home forcibly to us. And every year, as the League grows, and goes on from strength to strength, the importance of the occasion must be enhanced. Perhaps the occasion this year is not quite so important as next year's similar function will be, because, as I understand, next year this League will come of age, and I have special reason to congratulate the League most cordially on the Grand President under whom it will come of age. I might say this has always been a Prince of Wales' "show." It was started by a great Prince of Wales—King Edward—in the Diamond Jubilee year of Queen Victoria. I hope all the friends of the League will come forward, and induce others to come forward, and bring appropriate birthday presents, so that we may effectively wish the League "Many happy returns of the Day."

But there is another reason why this is a very special occasion. We are standing to-day between two worlds, one dead, the other waiting to be born. I will not say, as is sometimes being said, that peace is upon us with all its horrors, but peace certainly is upon us, and peace brings its problems as well as war: she also has her victories to win, victories almost as difficult as those of war. They will still require self-denial and struggle.

How does the war leave the League? I am afraid the war must leave—and that is the saddest thing about it—a legacy of impaired health and broken physique, and there will be a very great demand indeed for all the medical care and assistance that can be provided, even more so than in the past. How is the League prepared to meet this, and how do the hospitals of the country stand in regard to meeting this call? I daresay many of you know, from the admirable little brochure—one of many which the League has issued, called "The League of Mercy and the War," what can be done. It is a very great credit to the League. And if you want to go further, I suggest you do what I have done. I wanted to know how the hospitals stood, and I consulted one of the very greatest living authorities, who has done so much for the League, Sir Henry Burdett. I looked up his well-known red book, "Hospitals and Charities," and I was interested to see in the preface of this new edition the remark that the book would have been more complete—I should scarcely have thought that was possible—if some of the schedules had not been, unfortunately, torpedoed: they were on the s.s *Andania*. We do not bombard hospital ships: we confine ourselves to the more useful and harmless method of bombarding the public for subscriptions, and that I hope we shall continue to do. What, then, are the main lessons of the war, as you find them there?

The first thing that the war revealed was the shortage of beds in the hospitals. Hospitals have been tried by this war very hard, and it is clear that they have come out wonderfully, because of the great resources, the kindly feeling, the energy and the generosity which the public have thrown into their effort to support them. But still, it has been brought out that there was a great shortage of hospital accommodation. Sir Henry Burdett says the provision of beds for the civilian population was revealed as often miserably inadequate, and he goes on to say that that provision which has been made must never be allowed to drop again to the pre-war level. I am sure we must hope and trust that that will be so. But if it is to be so, I do really think it is largely to this League of Mercy we must look. Why do I say that? Because of the fundamental truth, which has already been brought out very strikingly by the Treasurer and other speakers, that this League is a very democratic institution. We are now plunging into an era of democracy, and this organisation, though it was founded by a Monarch and has been so largely and so splendidly supported by the Royal Family, is essentially democratic. Its principle is not to seek and attract the large gifts, but rather the small gifts of the many, and give to these donors the assurance that even the smallest, the "widow's mite," will be devoted wholly to the object for which it was given. (Hear, hear.) That, I think, is the principle on which we must go in these days. We hear a great deal about "No conscription!" I do not think any party has a monopoly of a wish for no conscription, we all wish that the voluntary system can prove itself sufficient. But what we must have in these days certainly is a splendid, small, highly-trained, mobile army, and at the same time we must have behind it a nation in arms. And in this matter we must

**The League of Mercy and its Future**—(*continued*).

have the whole nation mobilised against disease, just as it was mobilised against the foe in battle. It is my best hope that you will get that through this democratic League of Mercy. I saw, in the "Times," I think it was, recently, that of all the pregnant sayings of the present Prime Minister, if such he still is—David Lloyd George—the one which struck our American friends most was that you cannot run an A1 Empire on a C3 population. That, undoubtedly, is a great and striking truth. How are we to convert a C3 into an A1 population? May I again be a little bold and venture on a *memoria technica*? I remember seeing, a good many years ago, a cartoon in which was portrayed a gentleman reading the list of stations on a London railway, and he was saying, "It is a curious thing, but all the stations on this 'ere line begin with a haitch: there's 'ackney, 'olborn, 'ammersmith, and so on." I will give you a *memoria technica* of the same kind. We want, I think, three things, and whatever Government we have, I hope we shall go determinedly for them: Hygiene, Housing, Hospitals. But I do not want to see the hospitals taken over by the State: as I have said, I want to see no conscription: I want to see the perpetuation of the voluntary system, and the whole nation mobilised behind the hospitals in their war against disease.

Well, ladies and gentlemen, we stand for Mercy, that beautiful word. I am sure you know—and Lady Tree could put it better than I can—its attributes. "The quality of mercy is not strained. It droppeth as the gentle rain from Heaven on the place beneath. It is twice blest; it blesseth him that gives and him that takes." So Shakespeare told us. But the *quantity* of mercy is sometimes a little limited, a little strained, and has to be strained: and I think we must try to increase that quantity. And there, again, I think this League can do so much. I am full of hope, I am very optimistic. A friend of mine was saying, at the club, "We have got dreadful times upon us: we do not know what the Government is going to be, and the taxation will be enormous. We have got through the war, it is true, but I am not sure we shall not be ruined with peace." I said, "Well, we have won the war, and that is a splendid thing, and the country which has come through that will also come through other things. I build upon the young generation. One of the finest things has been the splendid way in which the men and women of the land, and not only the young ones either, have come forward and given their gifts and their personal help, and especially, perhaps, in caring for the sick. You have noticed how hospitals for them have sprung up everywhere. Country mansions, splendid London houses, cottages, schools, working men's dwellings, all have been turned into hospitals. We have seen how the men and women —perhaps especially the women—have given their ministrations, headed by the first woman in the land. I am sure they understand, and that this generation understands, far better than those of the last or the previous generations do, what the problem is and how it can be met. And so I feel that if you will use all your efforts in this democratic League, especially when headed by the Prince of Wales, we have a wonderful year, and many wonderful years before us, and I hope the League will do not a little to lift the life, in this and in other matters, to the highest plane. We have the knowledge: we have heard to-day a great deal that is instructive and inspiring. Let us spread that light and knowledge, not only in London, but also in the provinces. I was delighted to hear what was said about the Royal county of Berkshire, and I was pleased to hear, too, that Cambridge has done so well. Nothing does us so much good as rivalry, and if Cambridge does well, I hope we shall go one better at Oxford, and so stimulate Cambridge to even greater efforts. I hope this League and other agencies will continue to support the great hospitals with expert doctors to whom the poor can be sent if necessary, and continue to send down grants, as I know it does to Oxford. Let us remember that the hospital is the poor man's nursing home.

I feel it to have been a very great privilege to have been allowed to say anything on behalf of this League, and may I end by congratulating all its friends, and especially you, my Lord Chairman, on the position which, from the statements which have been made, it now occupies?

